# Structural screening and molecular simulation identify potential ligands against the K700E hot spot variant and functional pockets of SF3B1 to modulate splicing in myelodysplastic syndrome

**DOI:** 10.1016/j.heliyon.2024.e32729

**Published:** 2024-06-10

**Authors:** Rolando García, Murat Atis, Andrew Cox, Prasad Koduru

**Affiliations:** a Department of Pathology, UT Southwestern Medical Center, Dallas, TX, USA; b Lyda Hill Department of Bioinformatics, UT Southwestern Medical Center, Dallas, TX, USA

## Abstract

Myelodysplastic syndrome (MDS), a blood disorder with ineffective hematopoiesis and risk of transformation to acute myeloid leukemia, is characterized by recurring cytogenetic and molecular alterations. By chromosome analysis, approximately 60% of patients, carry chromosome 5 and 7 alterations, trisomy of chromosome 8 and may also present with increasingly complex karyotypes, especially in higher grade MDS (MDS with refractory anemia and increased blasts type 1 and 2). Moreover, somatic pathogenic variants in genes associated with aberrant mRNA splicing are frequently mutated with SF3B1 the most frequently mutated. In the setting of SF3B1, the K700E hot-spot mutation is present in approximately 50% of cases. Since recent studies have highlighted modulation of functional dynamics in SF3B1 by mutant splicing factors, the objective of the study was to identify potential small molecule modulators against the frequently mutated RNA splicing factor SF3B1(K700E) and functional allosteric sites by using a molecular structure-based approach and a molecular dynamic simulation. To identify potential SF3B1 modulators, we collected a series of chemical compounds from the Zinc and Enamine database. An initial screen followed by further molecular analysis and simulation using the Schrödinger suite was performed. Parameters used to monitor the stability and binding of the protein-ligand complex included: RMSF, protein-ligand contacts, electrostatic, Van Der Waals forces and binding energies (MMGBSA). A 100-nanosecond simulation showed strong binding between selected compounds and key amino acid residues, including the mutation hot-spot K700E and functional allosteric amino acid residue R630. Ligand binding energies between compounds and key amino acid residues ranged from −50.67 to −58.04 kcal/mol. In brief, small molecule modulators show strong binding to SF3B1 suggesting these compounds may be used against cells harboring the K700E variant or to modulate splicing by targeting functional allosteric sites.

## Highlights

• The current study explored small molecule modulators that target the mutational hot spot K700E and other functional pockets in SF3B1 by a virtual screen and molecular dynamics-based approach.
• This study identified four compounds, two of these FDA approved drugs, that target the mutational hot spot K700E and functional amino acid residue R630 that may have the potential to modulate SF3B1 activity in myelodysplastic syndrome (MDS) and other neoplasms linked to SF3B1 splicing control.
• These results represent new opportunities in targeting mutational hot spots and functional pockets in SF3B1 that contribute to the splicing regulation.

## 1 Introduction

Myelodysplastic syndrome (MDS) is a blood disorder characterized by cytopenia, ineffective hematopoiesis, future risk of acute myeloid leukemia, and genetic aberrations for structural-functional abnormalities of hematopoietic stem cells. It is clinically and genetically heterogeneous disease, primarily affecting the elderly population with a median age of presentation at 70–71 years. The categorized morphological features seen are dysplastic features in one or more cell lines of the myeloid lineage and contain an increased number of blasts; however, the number of blasts is less than 20 % in the bone marrow or peripheral blood. In terms of genetic defects, chromosome aberrations are detected in up to 60 % of patients, notably chromosome 5 and 7 alterations, trisomy of chromosome 8 and presentation of complex karyotypes. With the advent of newer genetic technologies, recurrent somatic variants in a number of genes have been reported in up to 90 % of MDS patients [1]. Sequence variants in genes associated with aberrant mRNA splicing (e.g., *SF3B1*, *U2AF1*, *SRSF2*, *ZRSR2*, *DDX41*, *LUC7L2*) are frequently mutated (50–89 %) and are associated with early clonal events and disease development [[2], [3], [4]]. Of these, *SF3B1* is by far the most frequently mutated comprising 20–30 % of cases [1,5] and the K700E substitution variant accounts for the majority of SF3B1 pathogenic sequence variants in MDS, refractory anemia with ring sideroblasts, acute myeloid leukemia (AML), chronic myelomonocytic leukemia, breast and pancreatic cancer, as well as, uveal melanoma [3,[6], [7], [8], [9]]. Other commonly mutated genes include histone modification and DNA methylation genes (*ASXL1*, *EZH2*, *TET2*, *DNMT3A*, *IDH1* and *IDH2*) [10,11]. Current therapies of MDS involve lenalidomide for deletion (5q), hypomethylating agents and regenerative hematopoietic stem cells to deter the drastic effects of blood cytopenia. More recently, potential new agents for both low and high-risk MDS are under investigation. However, despite these efforts, there is a need to develop novel therapies that target splicing factor alterations, since these are the most frequently mutated. Currently, small molecule modulators such as FR901464, herboxidiene, pladienolide B and analogues are documented. One analogue of pladienolide B (E7107) showed high affinity to SF3B1 and exhibited potent cytotoxicity activity against tumor cells, however, failed clinical activity in a clinical trial [12,13]. Likewise, H3B-8800, another derivative of pladienolide B undergoing clinical trial (NCT02841540), demonstrated dose dependent modulation of SF3B1 [14]. However, in this instance, only a small percentage of patients (14 %) obtained hematologic improvement, and complete remission was not observed in these patients [4]. Despite the potential therapeutic effect of H3B-8800, it is unclear at the molecular level how it modulates SF3B1 since the amino acid residues contributing to the binding site are far from the K700E hot-spot [15]. Nonetheless, HB3-8800 seems to have preferentially lethality against cancer cells harboring the K700E variant [15]. In a similar manner, other potential allosteric pockets near the K700E have been identified that may be targeted to modulate SF3B1 [15]. Nonetheless, despite a clear understanding of the molecular mechanism, recent studies have highlighted the functional dynamics of SF3B1 influenced by small splicing factor modulators or oncogenic mutations [16,17]. Both crystal structures of SF3B1 and cryo-EM studies have described its remarkable structural flexibility taking both open and closed conformations. This open or close structural flexibility allows SF3B1 to recognize unique intron sequences, specifically the branch point sequences (BPS) within an intron. Likewise, pathogenic mutations located far from the BPS recognition sites can engage in “crosstalk signaling” and exert an influence on the functional dynamics of spliceosome activity [18]. Indeed, molecular simulation studies have shown mutations disrupt SF3B1 and mRNA interactions [19,20]. Therefore, we explored potential small molecule modulators against the often-mutated K700E and other potential SF3B1functional allosteric sites [15,21], by a molecular structure-based approach and molecular dynamic analyses.

## 2 Materials and methods

To identify potential SF3B1 modulators, we curated a selection of chemical compounds from the Zinc database, which is accessible at https://zinc.docking.org/. Given the extensive range of compounds in the database and the significant role molecular weight and the compound hydrophobicity or lipophilicity (LogP) play in drug development (where compounds exceeding a molecular weight of 500 Da and LogP greater than 5 exhibit poor absorption or permeation), we concentrated our efforts on a subset of compounds categorized as "Goldilocks – druglike compounds" with a molecular weight up to 350 Da and a logP of 2. In addition, a list of FDA approved compounds from the Enamine database was also used for analysis. The H3B-8800 compound in phase-1 clinical trials for SF3B1 modulation was used as a control. All compounds were converted to single pdbqt file formats for molecular screening and docking using Vina and MGL tools (available at https://vina.scripps.edu/). The SF3B1 protein was obtained from the Protein Data Bank (PDB: 5ZYA), and docking sites were selected, including H572, L575, V576, E579, T612, M613, D616, N619, Y623, V624, T627, R630, A631, and K700E, [15,21]. The K700E hot spot variant in SF3B1was prioritized due to its most frequent mutation occurrence. Additionally, other amino acid residues selected for docking demonstrate high druggability and scoring potential for modulating SF3B1 [6,15]. For the control, the amino acid residues for docking included Y36, V37, R38, R1074, V1078, Y1157, V1114, R1075, and V1110. Binding affinity scores generated by Vina of less than −7.0 kcal/mol were selected for further analysis. The binding affinity between SF3B1 and small molecule modulators was later described by pKd values (>5 were considered strong binding affinity). The KDEEP package in PlayMolecule (available at: https://playmolecule.com) was used for generating pKd values. Following the initial screening, a second docking iteration was conducted, which involved generating an additional library comprising of potential compounds previously identified along with their stereoisomers. A total of 1000 copies of each compound was attempted. The second library was docked using glide Schrödinger module (Schrödinger, LLC, New York, NY). The clusters generated from stereoisomers were evaluated using matrix distance plots, contacts to desired amino acid residues, and binding energies generated of the clusters for further molecular analyses. To validate the interactions of compounds with the SF3B1 protein, we proceeded with additional molecular dynamics (MD) simulations using the Schrödinger Desmond module. Molecular simulations, which typically involve high-performance computers calculating how molecules move and interact over time, were prepared, and set up using established methods. At first, simulations were carried out for 300 ns (ns). However, if compounds didn't exhibit convergence (i.e., alignment of ligand and the protein) by the end of the simulation, as indicated by the RMSD plots, an extended simulation of 500 ns was conducted. The protein-ligand structures were placed in water molecules to mimic their natural environment. The water box used was about 132 × 132 × 132 cubic angstroms in size. Sodium and chloride ions were added to balance the charge and create a salt concentration like that found in biological systems, around 0.15 mol/L. To simulate how the protein and ligand behave, a mathematical model called the OPLS_2005 force field was used. This model helps predict the movements and interactions of the molecules. The simulation started by heating the system to a temperature of 300 K to mimic the conditions inside a living organism. Then, a type of simulation called the NPT ensemble was used, which maintains constant temperature and pressure. The pressure was controlled at 1.01325 bars, and the simulation ran for either 300 or 500 ns. Free energies of non-bonded interactions (electrostatic and van der Waals forces) to evaluate stability of protein-ligand complex were determined by using the Desmond package. To estimate the ligand-binding affinities based on free energy calculations, the molecular mechanics energies combined with the Poisson-Boltzmann or generalized Born and surface area continuum solvation (MM/GBSA) was used [22]. The QuikPro within the Schrödinger suite was employed to evaluate the physically relevant descriptors and pharmaceutical features of the prospective compounds targeting the K700E and functional pockets of SF3B1 [23]. In addition, a toxicity profile of the compounds was generated by the Pro-Tox-II server [24].

## 3 Results

### 3.1 Initial virtual screen

A total of 230,000 compounds from the Zinc database and 1040 FDA approved compounds were collected for analysis. The initial virtual screen performed identified 15 potential compounds with affinity to SF3B1 hot-spots and functional amino acid residues in SF3B1 with docking scores less than −7 kcal/mol and binding affinity scores (pKd values) greater than or equal to 5. Table 1 shows the list of small molecules with potential to modulate SF3B1.

**Table 1:** Docking scores and binding affinities of potential small molecule modulators against hot-spot amino acid residues in SF3B1.

Compound	Docking Score (kcal/mol)	Binding Affinity(Pkd)	Amino Acid
H3B-8800 (Control)	−7.43	5.50	E622, H662, Y36
Icatibant	−7.7	12.9	K700E
Thymopentin	−8.3	7.9	K700E
Goserelin	−8.3	12.3	K700E
Entrectinib	−10.1	7.5	K700E
ZN 630123660	−7.7	6.2	R630
			K700E
ZN253530914	−11.9	8.9	K700E
ZN299817872	−9.3	9.9	K700E
ZN253530915	−9.1	8.8	K700E
ZN252480685	−10.7	9.6	K700E
ZN263583855	−8.9	8.41	K700E
ZN100045486	−9.4	6.7	K700E
ZN261492935	−9.9	5.06	K700E
ZN95353169	−7.2	6.4	R630, K700E
ZN8551963 (Diquafosol)	−8.9	8.5	R630, K700E
ZN708900121	−9.5	9.21	ASP 616, THR 627

### 3.2 Second screen of identified stereoisomer compounds

Following the initial screening, a second docking iteration was conducted of stereoisomers of the 15 compounds. A grand total of 6167 stereoisomers were produced and then docked at various potential sites, mainly the K700E substitution and amino acid residues with druggability potential [15] to pinpoint ligand docking locations based on clusters. Not all compounds were replicated to the desired extent of 1000 stereoisomers. Only Goserelin, Icatibant, ZN299817872, ZN252480685, ZN263583855, and ZN261492935 generated 1000 copies. The rest of the compounds collectively generated a total of 169 copies. Selected poses for further molecular analysis were determined by evaluating distance matrix plots of compounds in clusters, the contacts to desired amino acid residues and their binding energy (only those compound poses with 7 kcal/mol or greater were selected for further molecular evaluation). Priority was given to binding energy and amino acid contacts when evaluating stereoisomer clusters.

**Fig. 1:**
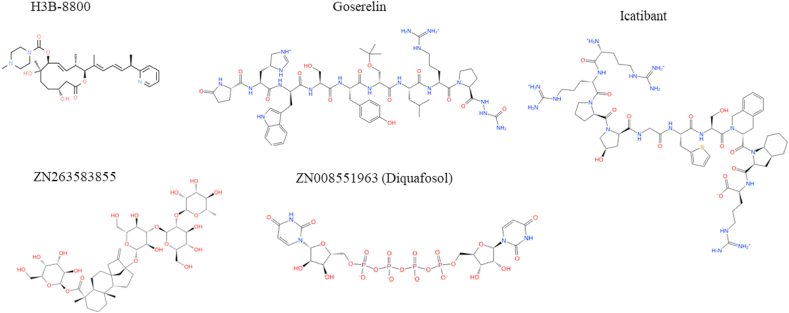
2-D Structures of the molecules selected for further molecular dynamic simulation.

Supplementary Fig. 1 illustrates the distance matrix plots of selected compounds, with the exception of ZN8551963-Diquafosol because only one stereoisomer copy was generated. Four compounds (Goserelin, Icatibant, ZN263583855 and ZN8551963- Diquafosol) were identified for subsequent molecular simulation analyses. Likewise, a simulation of the control compound (H3B-8800) was also performed. Fig. 1 illustrates the 2-D structures, while Table 2 describes the SMILE notation of compounds selected for molecular simulation.

**Table 2:** SMILE notation of ligands used for molecular simulation.

Compound	SMILE Notation
Control: H3B-8800	n1ccccc1[C@H](C)/C=C/C=C(\C)[C@@H](OC(=O)C[C@H](O)CC2)[C@@H](C)/C=C/[C@@H]([C@]2(C)O)OC(=O)N3CC[NH+](C)CC3
Goserelin	NC(=O)NNC(=O)[C@@H]1CCCN1C(=O)[C@H](CCCNC(N) = [NH2+])NC(=O)[C@H](CC(C)C)NC(=O)[C@@H](COC(C)(C)C)NC(=O)[C@H](Cc2ccc(O)cc2)NC(=O)[C@H](CO)NC(=O)[C@H](Cc3c[nH]c(*c*34)cccc4)NC(=O)[C@H](C[C@@H]5C[NH+] = CN5)NC(=O)[C@@H](N6)CCC6=O
Icatibant	CCNC(=O)[C@@H]1CCCN1C(=O)[C@H](CCCNC(N) = [NH2+])NC(=O)[C@H](CC(C)C)NC(=O)[C@@H](Cc2cn(c[nH+]2)Cc3ccccc3)NC(=O)[C@H](Cc4ccc(O)cc4)NC(=O)[C@H](CO)NC(=O)[C@H](Cc5c[nH]c(*c*56)cccc6)NC(=O)[C@H](Cc7c[nH+]c[nH]7)NC(=O)[C@@H](N8)CCC8=O
ZN263583855	C=C1C[C@]23CC[C@H]4[C@@](C)(CCC[C@@]4(C)C(=O)O[C@@H]4O[C@H](CO)[C@@H](O)[C@H](O)[C@H]4O)[C@@H]2CC[C@@]1(O[C@@, H]1O[C@H](CO)[C@@H](O)[C@H](O[C@@H]2O[C@H](CO)[C@@H](O)[C@H](O)[C@H]2O[C@@H]2O[C@@H](C)[C@H](O)[C@@H](O)[C@H]2O)[C@H]1O)C3
ZINC008551963 (Diquafosol)	O = c1ccn(c(=O)[nH]1)[C@H](O2)[C@H](O)[C@H](O)[C@H]2COP([O-])(=O)OP([O-])(=O)OP([O-])(=O)OP([O-])(=O)OC[C@@H]3[C@@H](O)[C@@H](O)[C@@H](O3)n(c(=O)[nH]4)ccc4 = O

### 3.3 Molecular simulation: ligand and protein RMSD

**Fig. 2:**
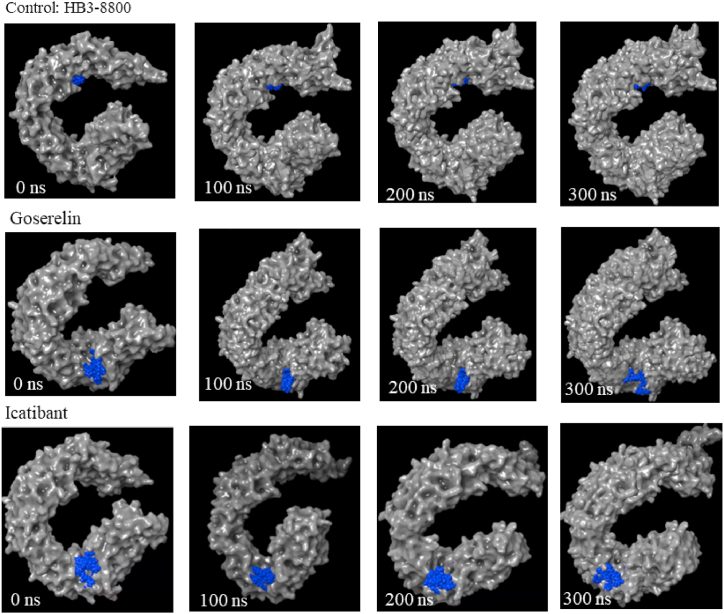
Molecular simulation of the H3B-8800 control and the two top compounds (painted in blue) bound to SF3B1 (highlighted in gray). The first 4 panels of each compound show the simulated trajectory (0–300 ns in 100 ns intervals). (For interpretation of the references to color in this figure legend, the reader is referred to the Web version of this article.)

A molecular simulation was initially conducted for a duration of 300 ns using the Schrödinger's Desmond package for the above-mentioned compounds, including the H3B-8800 control. Not surprisingly, the control attached to the same deep pocket as compound E7107 (both derivatives of pladienolide B), a pocket formed by amino acid residues Y36, V37, R38, R1074, R1075, V1078, V1110, V1114, V1078, and Y1157. Fig. 2 compares the binding of the control H3B-8800 and the top two compounds at different time intervals. The top two compounds (goserelin and icatibant) were selected based on the frequency of interactions with K700E, hydrogen bond contacts and energy binding energies to K700E.

**Fig. 3:**
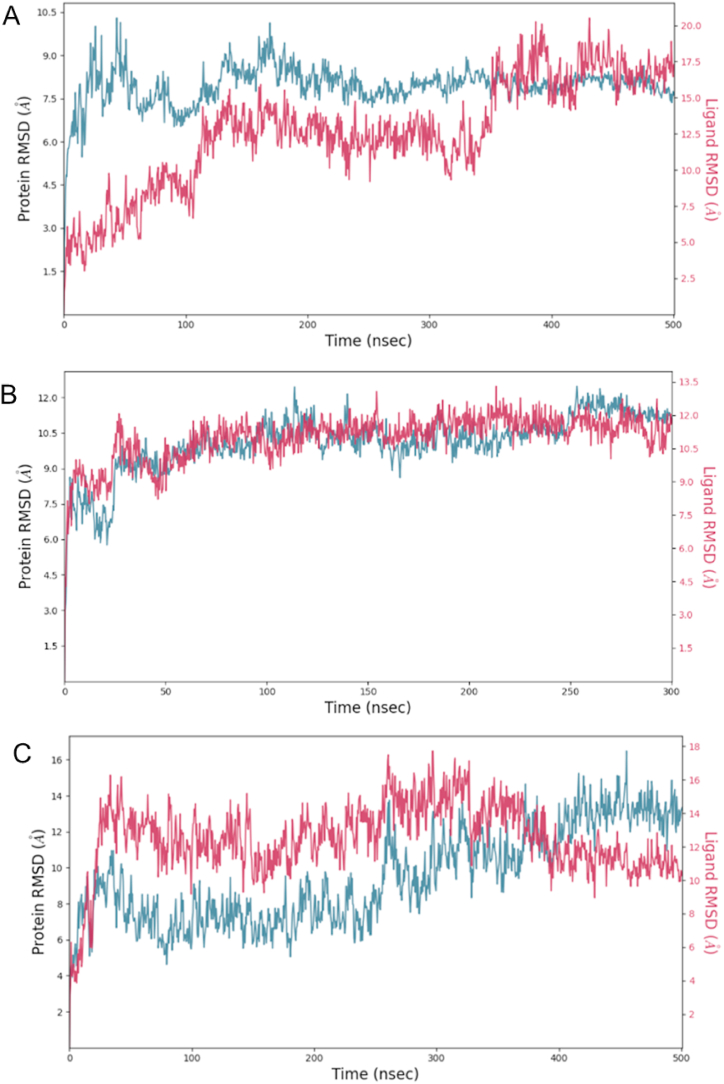

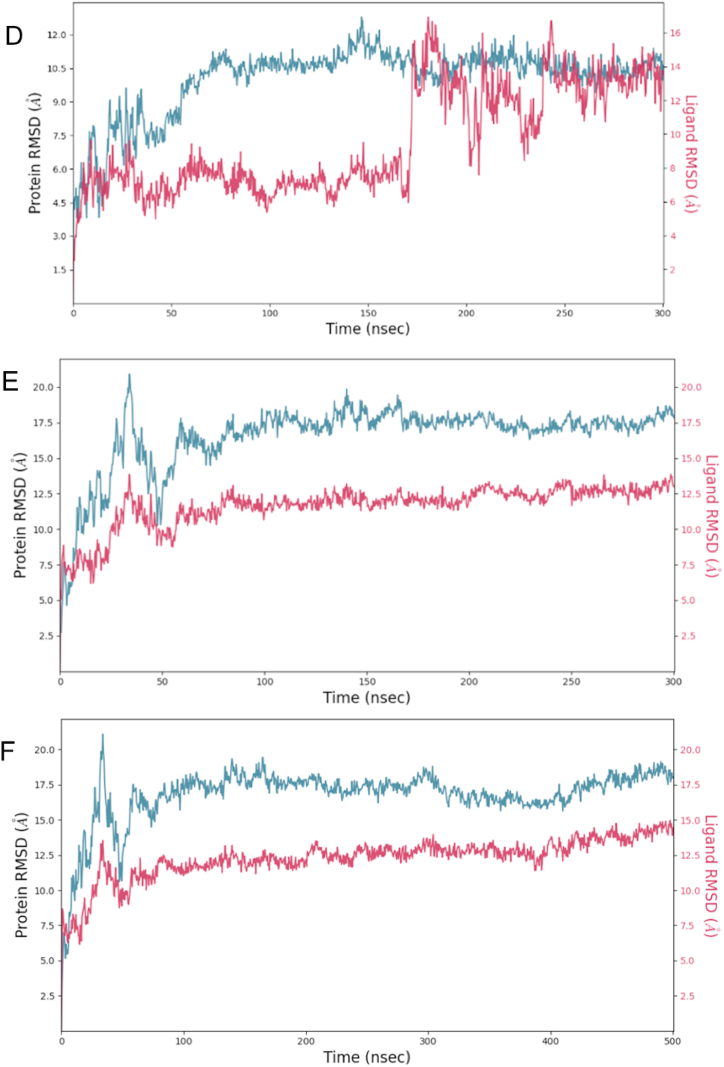
RMSD plots between SF3B1 and selected ligands. Panel A shows the control- H3B-8800 converged at approximately 350-ns. Panel B shows RMSD analysis of goserelin demonstrated a consistent superposition with the protein structure across the entire trajectory of 300-ns. This indicates goserelin maintained a stable binding conformation with the protein throughout the simulation. Panel C illustrates RMSD of the protein and icatibant over the course of 500 ns. Both the ligand and protein exhibited consistent stability throughout the simulation. However, some noticeable conformational changes occurred around 380 ns, although stability was restored shortly after 400 ns and persisted until the end of the simulation. Panel D illustrates RSMD convergence between ZN263583855 and SF3B1 at approximately 180-ns, but a more stable conformation of the protein and compound was reached approximately from 190 to 300-ns. Panels E and D depict RMSD plots for ZN8551963- Diquafosol and the protein at 300 and 500 ns, respectively. In this case, the protein and compound system did not achieve convergence. Nonetheless, they exhibited stable behavior throughout both simulation durations.

The evolution of both the ligand and the protein were tracked by monitoring the Root-mean-square deviation (RMSD) of the C-alpha atoms of the SF3B1 protein and ligand throughout the simulation. To assess the stability and binding of the protein-ligand complex, additional parameters such as Root-mean-square fluctuation (RMSF), protein-ligand contacts, electrostatic and van der Waals forces, and binding energies (evaluated using MM-GBSA) were also considered. The RMSD of the protein serves as a valuable indicator of structural conformation changes during the simulation (i.e., the structural stability of SF3B1 in the presence of the ligand), providing insights into equilibration status, while the ligand RMSD reflects the stability of the ligand with respect to the protein and binding pockets [25]. The convergence of ligand and compound indicates enhanced conformational stability of the protein-ligand complex. These implies that the system has reached a more stable state [26]. Moreover, conformational stability may also be noted with a stable behavior in RMSD supported by bonding to the same amino acid residues and binding energy calculations. Initially, a 300-ns molecular simulation of selected compounds was performed. If the compound and the protein did not converge at the end of this simulation, an extended time of 500-ns was performed (Fig. 3).

**Fig. 4:**
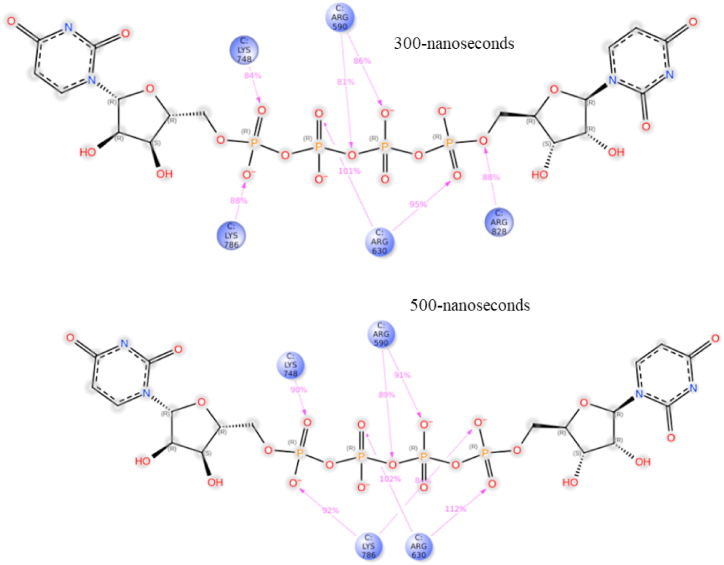
2-D structure of ZN8551963- Diquafosol bound to SF3B1 amino acid residues (colored circles) at two different time intervals (300 and 500-ns). The contact frequency for each of the amino acids with the ligand is given by the colored arrows.

Despite the lack of convergence between ZN8551963- Diquafosol and SF3B1, the consistent binding pattern observed throughout the 300 and 500-ns simulations, particularly involving residues R630, K786, K748, and R590, suggests enduring and robust nature of these interactions. This underscores the strength and specificity of the bonds formed between the ligand and the protein (Fig. 4).

### 3.4 RMSF of ligand and protein

The RMSF (root-mean square function) can be used to assess the molecular flexibility of protein binding pockets and ligand; it quantifies the movement of atoms and residues within a molecular structure. The lower the RMSF, the lower mobility of the binding site and the ligand, indicating a more favorable interaction and facilitating in the selection of potential drug candidates [27]. The RMSF values ranged from 1.65 to 3.65 $\overset{{}^{\circ}}{A}$. Amino acid residues with the most favorable interactions with the ligands (those with RMSF values in the range of 1–2 $\overset{{}^{\circ}}{A}$) included V1078, V1114, Y1157 in the control– H3B-8800; E622, R625, R630, H662, K666, K700E in goserelin and icatibant (Table 3). While ZN263583855- Diquafosol displayed slightly elevated RMSF values for certain amino acid residues, it is notable that specific atom numbers within the ligand, particularly oxygen 12 and 20, formed hydrogen bonds with the SF3B1 protein at R630, with distances of 2.40 Å and 2.12 Å, respectively.

**Table 3:** RMSF value measurements of ligand and amino acid residues.

Ligand/Amino acid residue	C-alpha/RMSF value - ($\overset{{}^{\circ}}{A}$)
Control: H3B-8800	
R1074	3.08
R1075	3.28
V1078	2.66
V1110	3.62
V1114	2.58
Y1157	2.69
Goserelin	
R630	2.12
K700E	2.16
Icatibant	
R630	2.72
K700E	3.02
ZN263583855 - Diquafosol	
R630	2.20
K700E	2.71
ZN008551963	
Y623	3.39
T627	3.41
R630	3.17
K700E	3.61

### 3.5 Contacts between SB3B1 protein and ligands

**Fig. 5:**
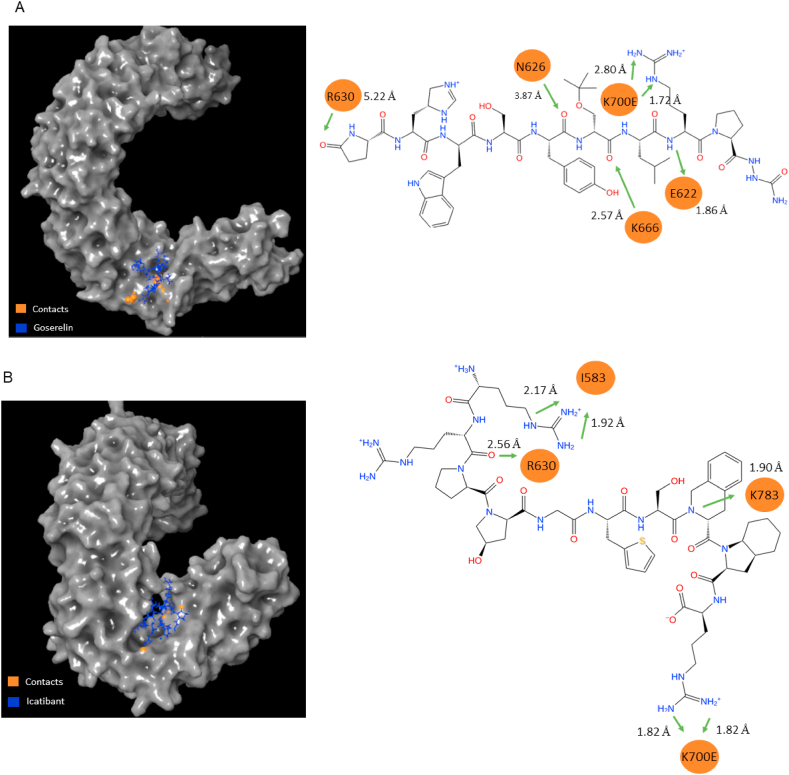
In Panels A & B, the illustrations demonstrate the intimate interactions and the specific amino acids engaged in the interactions between the selected ligands and SF3B1, notably the K700E and R630 hot spots. Hydrogen bonds at green arrows interact with amino acid residues (highlighted in orange), along with the corresponding distances between the ligand atoms and the amino acid residue. (For interpretation of the references to color in this figure legend, the reader is referred to the Web version of this article.)

Protein interactions with the various ligands were monitored during the simulation (Table 4). The protein-ligand interactions were divided into four categories: hydrogen bonds, hydrophobic interactions, ionic interactions, and water bridges (i.e., hydrogen bonded protein and ligand interactions mediated by water molecules that are slightly more relaxed that the standard hydrogen bond definition). Note that interaction values greater than 100 % (1.0) can occur when certain protein residues engage in multiple contacts with the ligand. For example, goserelin formed two hydrogen bonds with K700E; ZN263583855- Diquafosol and ZN008551963 formed two hydrogen bonds each with R630. Among the selected ligands with the highest frequency of contacts to K700E (greater than 100 %) included goserelin (1.85) and icatibant (1.13). Fig. 5 shows the close interaction of goserelin and icatibant with SF3B1 amino acid residues, especially the K700E and R630 residue hot spots.

**Table 4:** Protein-Ligand Contacts (hydrogen bonds, hydrophobic bonds, ionic and water bridge bonds) to amino acid residue hot spots expressed as a percentage.

Compound/Amino Acid Residue	Hydrogen	Hydrophobic	Ionic	Water Bridge	Total
Control H3B-8800					
R1074	0.01	0	0	0.14	0.15
R1075	0	0	0	0.01	0.01
V1078	0	0.03	0	0	0.03
V1110	0	0.03	0	0.02	0.05
V1114	0	0.23	0	0	0.23
Y1157	0.11	0.62	0	0.10	0.83
Goserelin					
R630	0.55	0.12	0.01	0.32	1.00
K700E	1.63	0	0.08	0.14	1.85
Icatibant					
R630	0.01	0	0	0.10	0.11
K700E	0.70	0	0.04	0.39	1.13
ZN263583855					
R630	1.20	0	0.01	0.21	1.42
K700E	0.03	0	0	0.37	0.40
ZN008551963					
Y623	0	0	0	0.21	0.21
T627	0.01	0	0	0.42	0.43
R630	2.08	0	0.16	1.89	4.13
K700E	0	0	0	0.07	0.07

### 3.6 Free energy calculations

We also conducted free energy calculations (non-bonded interactions: electrostatic and van der Waals forces) to assess conformation stability of protein-ligand complex throughout the trajectory. Calculations derived from MM-GBSA were also used to assess the strength of the ligand-receptor interactions [28]. Indeed, the higher the free energy, the more stable and higher affinity, this means that protein-ligand interactions occur spontaneously and release of more energy. Table 5 displays the cumulative energy, electrostatic and van der Waals forces, calculated for protein-ligand complex for the different compounds (control H3B-8800, goserelin, Icatibant, ZN26358385 and ZN008551963-Diquafosol at four distinct time intervals (0, 100, 200, and 300 ns). The free energies of compounds extended to 500 ns are also included. Moreover, MM-GBSA free energy calculation of ligands and SF3B1 amino acid residues was performed(see Table 6).

**Table 5:** Energies of protein-ligand at various time intervals.

H3B-8800 (control)				
Time (ns)	Elec	VdW	Nonbond	Total (kcal/mol)
0	−80.39	−34.07	−114.46	−114.46
100	−23.50	−29.45	−52.95	−52.95
200	−70.86	−32.35	−103.21	−103.21
300	−110.15	−41.12	−151.27	−151.27
500	−96.21	−21.83	−118.04	−118.04

Goserelin				
0	−118.84	−48.40	−167.24	−167.24
100	−297.67	−35.96	−333.63	−333.63
200	−279.27	−23.34	−302.61	−302.61
300	−249.27	−43.58	−292.85	−292.85

Icatibant				
0	−228.58	−35.38	−263.96	−263.96
100	12.88	−37.06	−24.18	−24.18
200	−68.31	−28.14	−96.45	−96.45
300	−93.84	−27.59	−121.43	−121.43
500	−221.88	−44.51	−266.39	−266.39

ZN263583855				
0	−139.01	−48.36	−187.37	−187.37
100	−55.89	−35.84	−91.73	−91.73
200	−24.72	−19.16	−43.88	−43.88
300	−50.88	−37.28	−88.16	−88.16

ZN008551963-Diquafosol				
0	−373.01	−22.55	−395.56	−395.56
100	−683.65	−25.26	−708.91	−708.91
200	−732.04	−23.83	−755.87	−755.87
300	−746.82	−15.02	−761.84	−761.84

Key: ns, nanoseconds, Elec, electrostatic interactions; VdW, van der Waals interactions; Nonbond, nonbonded.

**Table 6:** Ligand binding energies to SF3B1 amino acid hot spot residues at convergence or noted behavior stability.

Compound	Amino acid Residue	Binding Energy (Kcal/mol) MM-GBSA
Goserelin	R630	−76.08
	K700E	−183.71
Icatibant	R630	34.99
	K700E	−161.91
ZN263583855	R630	−57.75
	K700E	−164.28
ZN008551963-Diquafosol	Y623	−41.35
	T627	−34.49
	R630	−110.78
	K700E	−96.31

Note that MM-GBSA calculations demand substantial time and computational resources, especially when handling complete simulations. It's important to recognize that the resulting binding energy from MM-GBSA reflect calculations observed at the end of the simulation. This typically occurs when both the ligand and protein RSMD have converged, or a noted behavior stability in their interaction is observed, such in the case of ZN8551963- Diquafosol and SF3B1. In contrast, metrics like RMSD (refer to Fig. 2), protein-ligand contacts (see Table 4), and total non-bonded energy calculations (detailed in Table 5) offer insights spanning the entirety of the simulated trajectory.

Considering the above molecular analyses and binding energies, the four selected compounds demonstrated affinity interactions and free energy binding to the hot spot variant K700E and functional hotspots. Specifically, goserelin exhibited both an increased number of interactions and stronger binding affinity to K700E compared to other functional hotspots. Meanwhile, ZN008551963-Diquafosol showed a preference for binding functional pockets, particularly R630.

### 3.7 Drug-like properties and toxicity assessment of ligands

Predictions of drug-like properties (properties found in 95 % of pharmaceutical compounds) of the selected ligands were derived from the QuikPro property predictions module in Schrödinger. Our analysis included the following properties: molecular weight, dipole moment, total SASA (total solvent accessible surface area), hydrophilic and hydrophobic SASA, number of donor and acceptor hydrogen bonds, molecular volume, globularity, solute ionization potential, QP polarizability, QP log P for hexadecane/gas, QP log S for aqueous solubility, QP log BB (brain-blood partition coefficient), QPlogKhsa (human serum albumin binding) number of primary metabolites, Apparent Caco-2 Permeability, apparent MDCK cell permeability, Lipinski violation (rule of 5), and the Jorgensen's rule of three. The predictions of these features are documented in the Supplementary Table 1. Based on QuikPro property prediction calculations, the larger molecular weight FDA approved compounds (>725 atomic mass units) icatibant and goserelin, along with Zinc compounds, ZN263583855, and ZN008551963- Diquafosol, had more unsatisfactory ranges, while the smaller molecular weight control H3B-8800 (molecular weight of 555) exhibited more acceptable ranges. It's important to note that QuikPro property predictions, which encompass properties observed in 95 % of therapeutic compounds, are valuable but not absolute determinants. Comprehensive evaluation of compounds as effective therapeutics should also take into consideration additional factors such as clinical data, risk-benefit analysis, and therapeutic necessity. In addition to evaluating properties frequently observed in 95 % of therapeutic compounds, we conducted a toxicity assessment using probability derived calculations from the Pro-Tox-II server. Here, 12 toxicity metrics were evaluated: Predicted LD50 (Dose value), hepatotoxicity, neurotoxicity, respiratory toxicity, cardiotoxicity, immunotoxicity, cytotoxicity, blood brain barrier, ecotoxicity, clinical toxicity, mutagenicity, and cytotoxicity. Although all the compounds, including the control showed some degree of predicted toxicity (painted circles in red, see Table 7), all were classified as either moderate or non-toxic based on predicted toxicity classification. However, note that further assessment and validation studies, including in-vitro and in-vivo studies, are needed to confirm the toxicity potential predicted by computational methods. These studies can provide better information into the effects of these compounds.

**Table 7:**
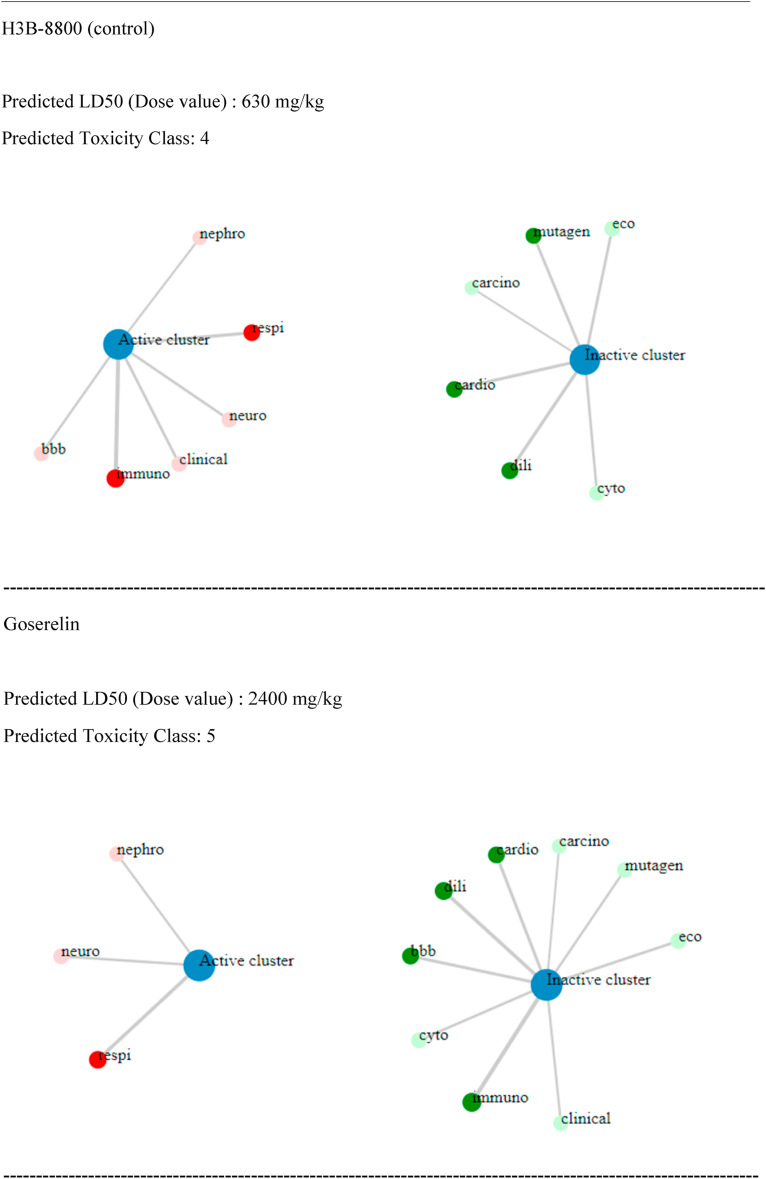

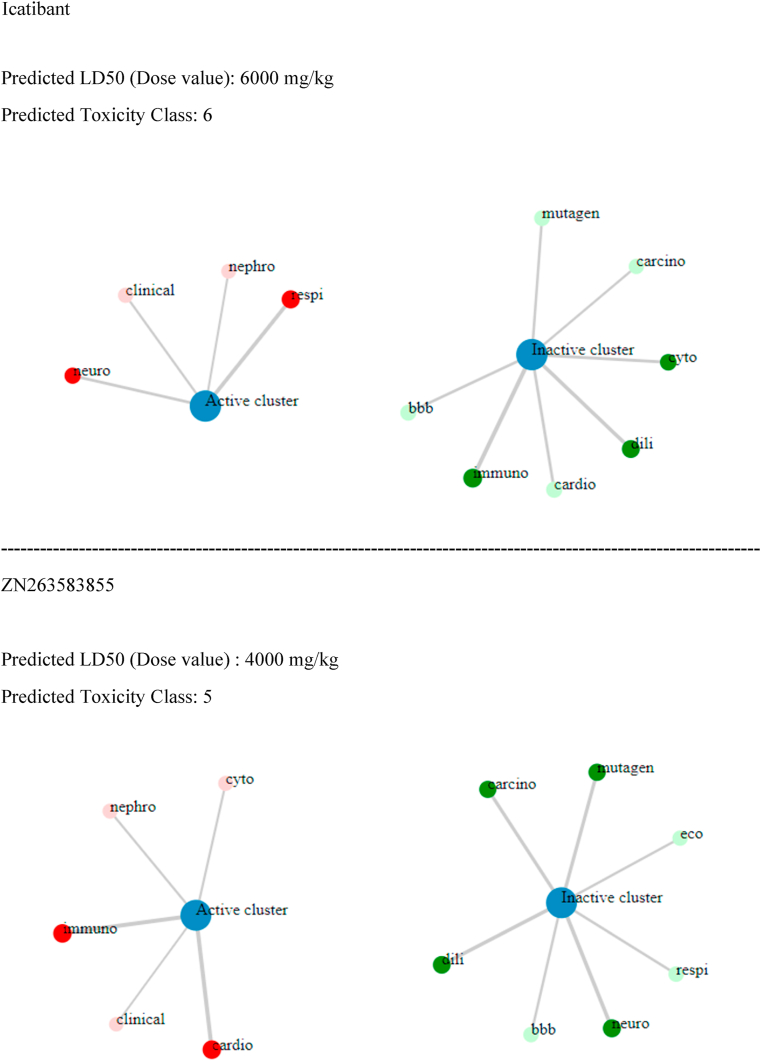

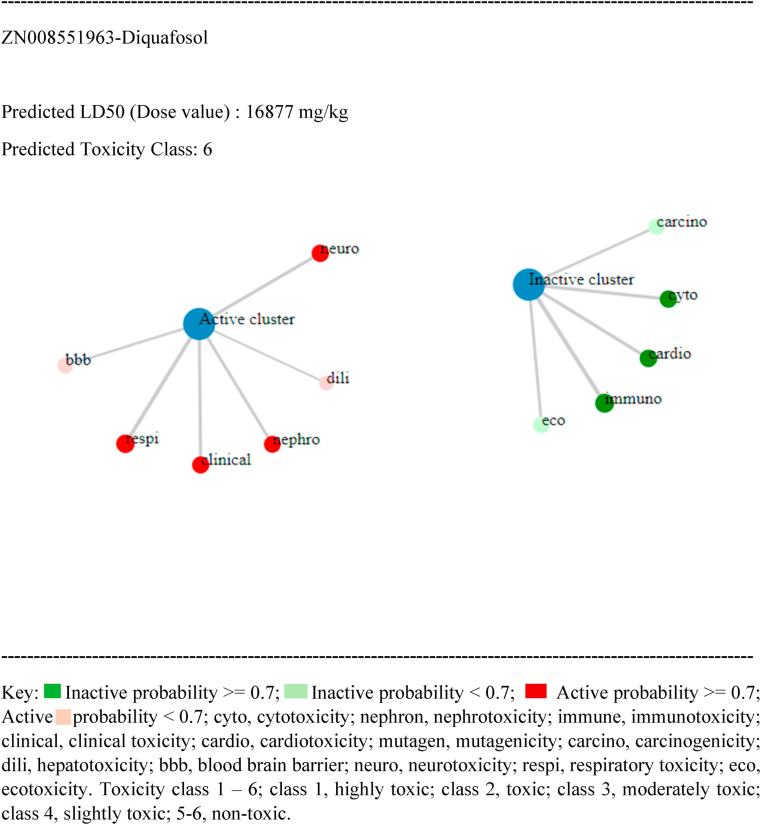
Toxicity Profile of selected compounds.

## 4 Discussion

In this structure-based approach, combined with molecular dynamics, we have introduced four potential compounds (Goserelin, Icatibant, ZN263583855 and ZN008551963- Diquafosol) designed to target the K700E hot spot variant and functional pockets, mainly R630 within SF3B1.

The observed characteristics of these compounds in molecular dynamics simulations indicate their promising potential to selectively modulate or remove the aberrant splicing activity of K700E mutated SF3B1. This suggests that they could have a notable impact on the treatment of MDS, but also extends to a broader spectrum of clinical implications in other cancer types associated with the SF3B1 K700E mutation, underscoring the significance and widespread applicability of our findings. Other conditions with SF3B1 K700E include acute myeloid leukemia (AML), occurring in 2–5% of AML cases, chronic lymphocytic leukemia (CLL) observed in 15 % with poor prognosis, uveal melanoma in 15 %, pancreatic ductal adenocarcinoma (PDAC) in 4 % where K700E is an oncogenic driver, and 16 % in papillary breast cancers [9,[29], [30], [31]]. These conditions present opportunities for exploration and potential therapeutic avenues, by addressing the underlying K700E abnormality and modulating SF3B1 activity. In patients with MDS, SF3B1 mutations are prevalent (approximately 25 % in all MDS cases) and occurring in approximately 85 % of cases in a form of MDS (refractory anemia with ringed sideroblasts) [32], with K700E the most frequently observed mutation. The presence of K700E in myeloid progenitors induces abnormal splicing patterns, reflecting the phenotype observed in MDS. This suggests that targeting this aberration could hold promise as a potential therapeutic strategy for MDS. The strong affinities of Goserelin, Icatibant and ZN26358385 for the K700E hot spot suggest that these compounds may be suitable for targeted therapies in these neoplasms. In addition, these compounds may be used in combination therapies, potentially enhancing their therapeutic effects, and overcoming resistance mechanisms. Similarly, the identification of strong binding to the functional allosteric residue R630, mainly ZN008551963-Diquafosol, indicates that the compounds may exert effects through allosteric modulation of protein function [15]. This could open new avenues for therapeutic intervention, particularly if targeting this residue can disrupt aberrant splicing and signaling pathways or enhance the efficacy of existing therapies in MDS or other cancer types with this mutation. Nevertheless, further research, including in-vitro and in-vivo validation into the therapeutic potential of these compounds is needed to fully assess the therapeutic potential of these compounds and determine their applicability in clinical settings.

Currently, the predominant method of manipulating SF3B1 in tumor cells relies on small molecules [33], a strategy that has shown promise in various diseases, including cancer. Computational methods have emerged as invaluable tools in this pursuit, streamlining the process of identifying lead compounds and accelerating their path to market. A vast number of compounds identified through these methods have already made significant strides in treating diseases such as viral infections and cancer, including HIV inhibitors, tyrosine kinase inhibitors for chronic myeloid leukemia, and ALK inhibitors for pancreatic cancer, among others [[34], [35], [36], [37]]. In terms of SF3B1, a list of potential SF3B1 modulators is reported in the literature, with compounds like H3B-8800, spliceostatin A, and E7107 demonstrating promising outcomes in preclinical studies [[38], [39], [40]]. These compounds exhibit efficacy across a spectrum of solid tumors and hematological neoplasms, offering hope for patients with MYC-driven cancers, metastatic neuroblastoma, glioblastoma multiforme, and various others [38,39,41,42]. However, challenges have surfaced during clinical trials, as seen with E7107, which was discontinued due to vision impairment concerns [43]. H3B8800, a splicing modulator and synthetic derivative of pladienolide, enter clinical trials (NCT02841540) for patients with MDS, acute myeloid leukemia and chronic myelomonocytic leukemia. Despite splicing modulation activity and reduced transfusions in patients, H3B-8800 did not show any clinical response [14,44]. Therefore, additional efforts are needed to identify and to test additional compounds to modulate the splicing activity of SF3B1. Recent studies have shed light on potential mechanisms underlying the cytotoxic effects of these compounds, including cell cycle regulation, apoptosis induction, and DNA double-stranded break induction [45,46]. However, further research is needed to obtain a better understanding of the mechanisms through which SF3B1 modulators induce anti-proliferative effects in tumor cells. Considering the advantages and benefits associated with conducting small molecule screens and their potential in identifying possible SF3B1 modulators targeting tumor cells, we conducted a review of the literature of the four compounds identified as potential modulators in this study. To the best of knowledge, there is limited available data for ZN263583855 (searchable under the Zinc database as ZINC00263583855). The remaining three compounds are FDA approved, with ZN008551963- Diquafosol used as a dry eye treatment, Icatibant used in hereditary angioedema, viral infections, and sweating induced dermal pain, and Goserelin, use to treat breast and prostate cancer [[47], [48], [49], [50], [51]]. The latter compounds hold a promise for the repurposing as SF3B1 modulators.

### 4.1 Limitations and future work

Although these results show promising results, our molecular structure-based approach may face limitations. For instance, certain biological or physical processes may extend beyond the duration of 300 or 500 ns, restricting our understanding of system behavior and requiring additional computational resources such as time and computational power. However, our observations of the time scales spanning 300 and 500 ns suggested both protein and ligand reached a state of equilibrium within the system for three of the four compounds. One compound, ZN008551963- Diquafosol did not achieve equilibrium but did exhibit consistent stability throughout the trajectory of 300 and 500 ns. Despite limitations in long timescale dynamics, the use of molecular simulations offers valuable insights into the behavior of compounds at the molecular level, aiding in the discovery and development of novel therapeutics. Moving forward, several avenues of future research include in-vitro and in-vivo validation studies, exploring molecular mechanisms underlying the interaction between the ligands and SF3B1 through mechanistic investigations to better understand their mode of action and potential therapeutic targets, optimization of the identified ligands through chemistry approaches that may improve their potency, selectivity, and pharmacokinetic properties, and structure-activity relationship studies with analog synthesis that may lead to the development of more effective SF3B1 modulators with enhanced therapeutic potential.

## 5 Conclusion

Our research study has identified four ligands exhibiting molecular features that hold promise in regulating SF3B1 activity within MDS and potentially other neoplasms associated with the SF3B1 K700E mutation. These ligands target the mutant hot-spot K700E as well as the allosteric functional site R630. These findings represent a step forward in the development of potential therapeutic interventions in these patients. While further research and validation is needed, identification of these compounds may open new avenues for targeted therapies that may ultimately improve the quality of life of individuals affected by MDS and beyond.

## Institutional review board statement

Not applicable.

## Data and availability statement

Data included in article/supplementary material/referenced in article.

## CRediT authorship contribution statement

**Rolando García:** Writing – original draft, Visualization, Software, Project administration, Methodology, Investigation, Formal analysis, Data curation, Conceptualization. **Murat Atis:** Formal analysis, Data curation. **Andrew Cox:** Formal analysis. **Prasad Koduru:** Writing – review & editing, Supervision.

## Declaration of competing interest

The authors document no competing interests or personal relationships that may have influenced the work of this study.
