# Supplementary material for: Structural screening and molecular simulation identify potential ligands against the K700E hot spot variant and functional pockets of SF3B1 to modulate splicing in myelodysplastic syndrome
Source: Heliyon. 2024 Jun 10;10(12):e32729. doi: 10.1016/j.heliyon.2024.e32729 (PMC11225765; doi:10.1016/j.heliyon.2024.e32729)
Supplement: Multimedia component 1 [file mmc1.docx]

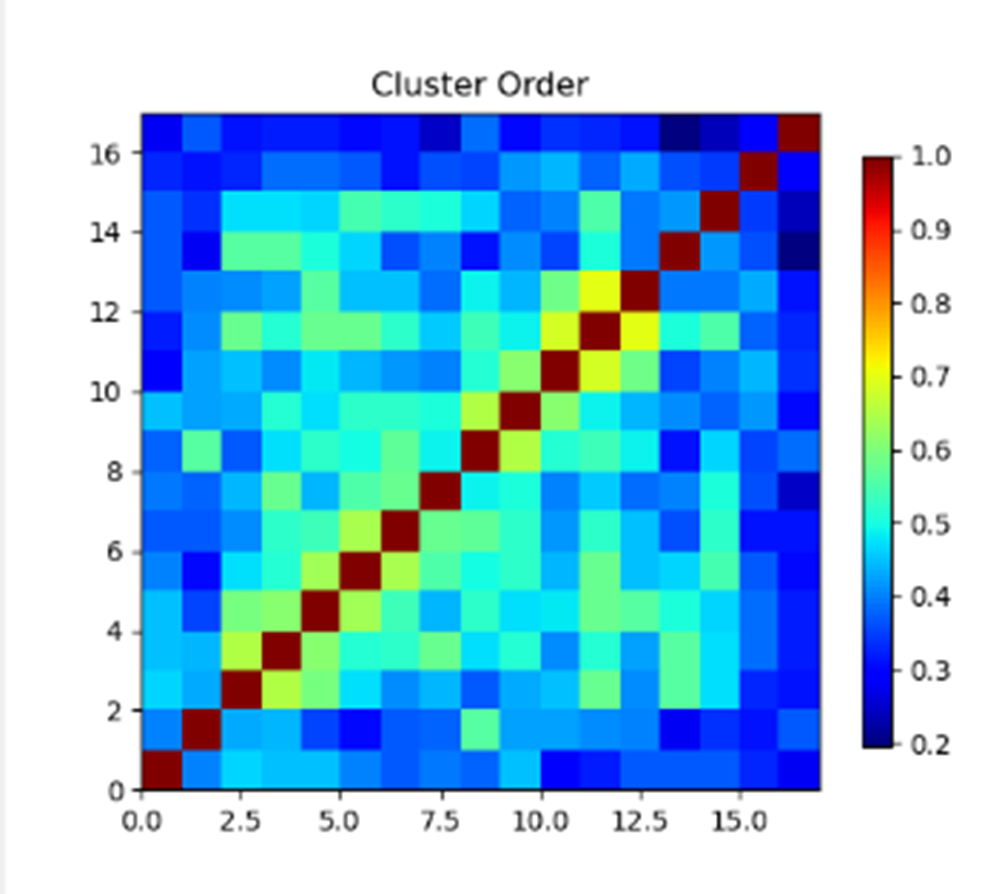

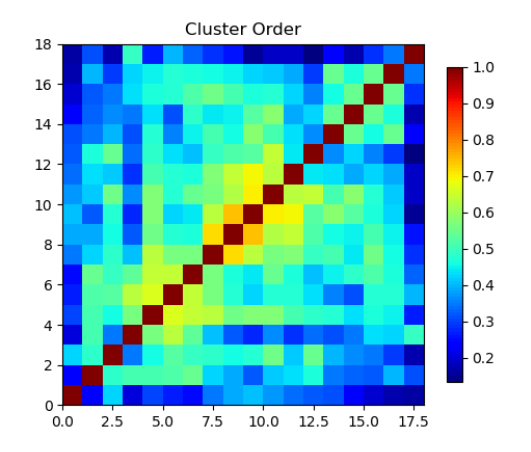


Goserelin (n = 1000, 5 clusters) Icatibant (n = 1000, 5 clusters)

16 bound stereoisomers depicted. 18 bound stereoisomers illustrated.


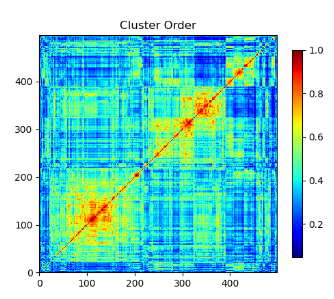

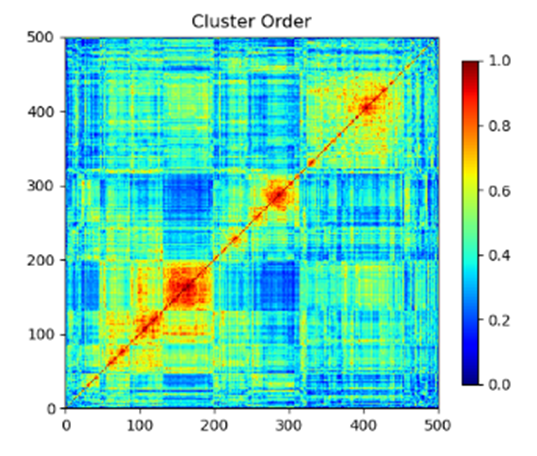


ZN263583855 (n = 500, 12 clusters) ZN263583855 (n = 500, 28 clusters)

Supplemental Figure 1. Distance matrix plots depict clusters formed by selected compounds. Cluster regions highlighted in red, orange indicate the highest similarity scores between clustered compounds. Note that although goserelin and icatibant had a total of 1000 generated stereoisomers each, only a limited number of copies displayed binding affinity and clustering to the variant amino acid residue K700E and functional pockets. Compound ZN263583855 was split into two halves (n = 500 each) to optimize computational processing time and resource utilization. The stereoisomer of each compound selected for further molecular analysis was based on binding affinity, amino acid contacts and clustering-distance matrix plots (i.e., high similarity score of stereoisomers). Binding energy and amino acid contacts were given priority for additional molecular analysis.

Supplementary Table 1. ADMEN/TOX (For 95% of known drugs.)

**______________________________________________________________________________**

Compound / Descriptor Range Value

______________________________________________________________________________

**Control (H3B-8800)**

**Drug-Like Descriptors (Range of 95% of drugs)**

Molecular Weight (130-725) 555.713

Dipole Moment (1-12.5) 3.276

Total SASA (300-1000) 954.881

Hydrophilic SASA (7-330) 144.354

Hydrophobic SASA (0-750) 624.220

Donor H-bond (0 -6) 1

Acceptor H-bond (2-20) 8.95

Molecular Volume (500-2000) 1818.207

Globularity (0.75-0.95) 0.7544747

Solute Ionization (7.9-10.5) 8.957

Potential

**Prediction for Properties**

QP Polarizability (13-70) 63.895M

QP log P for (4.0-18.0) 17.949M

hexadecane/gas

QP log S for aqueous solubility (-6.5 – 0.5) -7.802M*

QP log K hsa (-1.5 – 1.5) 1.427M

QP log BB (-3.0 – 1.2) -1.151M

No. of Primary Metabolites (1.0-8.0) 11*

Apparent Caco-2 Permeability (<25 poor, >500 great) 105

Apparent MDCK Permeability (<25 poor, >500 great) 48

Lipinski Violation (1-5) 2

Jorgensen’s rule of three (1-3) 2

**---------------------------------------------------------------------------------------------------------------------**

**Goserelin**

**Drug-Like Descriptors (Range of 95% of drugs)**

Molecular Weight 1269.425*

Dipole Moment 14.645*

Total SASA 1726.319*

Hydrophilic SASA 615.741*

Hydrophobic SASA 809.918*

Donor H-bond 11.5*

Acceptor H-bond 25.7*

Molecular Volume 3592.320*

Globularity 0.657*

Solute Ionization 8.173

Potential

**Prediction for Properties**

QP Polarizability 117.366M*

QP log P for 42.106M*

hexadecane/gas

QP log S for aqueous solubility -3.438M

QP log K hsa -2.510M*

QP log BB -11.114M*

No. of Primary Metabolites 18*

Apparent Caco-2 Permeability 0.001*

Apparent MDCK Permeability 0*

Lipinski Violation 3

Jorgensen’s rule of three 2

**---------------------------------------------------------------------------------------------------------------------**

**Icatibant**

**Drug-Like Descriptors (Range of 95% of drugs)**

Molecular Weight 1304.532*

Dipole Moment 13.428*

Total SASA 1830.939*

Hydrophilic SASA 778.172*

Hydrophobic SASA 770.306*

Donor H-bond 17.250*

Acceptor H-bond 32.150*

Molecular Volume 3667.152*

Globularity 0.628*

Solute Ionization 8.177

Potential

**Prediction for Properties**

QP Polarizability 119.777M*

QP log P for 46.449M*

hexadecane/gas

QP log S for aqueous solubility -4.445M

QP log K hsa -3.946M*

QP log BB -14.119M*

No. of Primary Metabolites 24*

Apparent Caco-2 Permeability 0*

Apparent MDCK Permeability 0*

Lipinski Violation 3

Jorgensen’s rule of three 2

**---------------------------------------------------------------------------------------------------------------------**

**ZN263583855**

**Drug-Like Descriptors (Range of 95% of drugs)**

Molecular Weight 951.02*

Dipole Moment 4.50

Total SASA 1236.03*

Hydrophilic SASA 501.51*

Hydrophobic SASA 709.01

Donor H-bond 13*

Acceptor H-bond 35.05*

Molecular Volume 2574.70*

Globularity 0.73

Solute Ionization Potential 9.78

**Prediction for Properties**

QP Polarizability 81.72*

QP log P for 28.81*

hexadecane/gas

QP log S for aqueous solubility -2.38

QP log K hsa -2.27*

QP log BB -7.07*

No. of Primary Metabolites 14*

Apparent Caco-2 Permeability 0.17*

Apparent MDCK Permeability 0.043*

Lipinski Violation 3

Jorgensen’s rule of three 2

**---------------------------------------------------------------------------------------------------------------------**

ZN008551963- Diquafosol

**Drug-Like Descriptors (Range of 95% of drugs)**

Molecular Weight 790.31*

Dipole Moment 7.61

Total SASA 934.51

Hydrophilic SASA 688.39

Hydrophobic SASA 148.78

Donor H-bond 6

Acceptor H-bond 33*

Molecular Volume 1796.86

Globularity 0.76

Solute Ionization 9.47

Potential

**Prediction for Properties**

QP Polarizability 53.76

QP log P for 21.98*

hexadecane/gas

QP log S for aqueous solubility 0.39

QP log K hsa -3.87*

QP log BB -8.47*

No. of Primary Metabolites 6

Apparent Caco-2 Permeability 0*

Apparent MDCK Permeability 0*

Lipinski Violation 3

Jorgensen’s rule of three 1

**---------------------------------------------------------------------------------------------------------------------**

An (*) indicates a violation of the 95% range.
